# A sex-role-preference model for HIV transmission among men who have sex with men in China

**DOI:** 10.1186/1471-2458-9-S1-S10

**Published:** 2009-11-18

**Authors:** Jie Lou, Jianhong Wu, Li Chen, Yuhua Ruan, Yiming Shao

**Affiliations:** 1 Department of Mathematics, Shanghai University, 99 Shangda Road Shanghai, 200444, PR China; 2 MITACS Centre for Disease Modeling, York University, Toronto, Ontario, Canada M3J 1P3; 3 Department of Research on Virology and Immunology, National Center for AIDS/STD Control and Prevention, 27 Nanwei Road, Beijing, 100050, PR China

## Abstract

**Background:**

Men who have sex with men (MSM) are much more likely to be infected with HIV than the general population. China has a sizable population of MSM, including gay, bisexual men, money boys and some rural workers. So reducing HIV infection in this population is an important component of the national HIV/AIDS prevention and control program.

**Methods:**

We develop a mathematical model using a sex-role-preference framework to predict HIV infection in the MSM population and to evaluate different intervention strategies.

**Results:**

An analytic formula for the basic reproduction ratio *R*_0 _was obtained; this yields *R*_0 _= 3.9296 in the current situation, so HIV will spread very fast in the MSM population if no intervention measure is implemented in a timely fashion. The persistence of HIV infection and the existence of disease equilibrium (or equilibria) are also shown. We utilized our model to simulate possible outcomes of antiretroviral therapy and vaccination for the MSM population. We compared the effects of these intervention measures under different assumptions about MSM behaviour. We also found that *R*_0 _is a decreasing function of the death rate of HIV-infected individuals, following a power law at least asymptotically.

**Conclusion:**

HIV will spread very fast in the MSM population unless intervention measures are implemented urgently. Antiretroviral therapy can have substantial impact on the reduction of HIV among the MSM population, even if disinhibition is considered. The effect of protected sexual behaviour on controlling the epidemic in the MSM population largely depends on the sex-ratio preference of different sub-populations.

## Background

The report from the American Foundation for AIDS Research [1] suggests that the group originally at the most risk of HIV - gay and bisexual men - still remains at the highest risk. This is largely due to anal sex which, when unprotected, carries a high risk of HIV transmission, especially for the receptive partner. Men who have sex with men (MSM) are 19 times more likely to be infected with HIV than the general population. Gay and bisexual men are only a part of the total MSM population, since MSM is a description of a behavioural phenomenon, not an identity.

China's first, and most recent, official figure on male homosexuality was released in 2004, putting the total of gay men in the country at between five and ten million [2]. But this is only a conservative estimation [3]. The HIV infection rate among gay men in China is climbing at an alarming rate, largely due to neglecting this subpopulation. Recent studies suggest unprotected risk behaviour or sexually transmitted diseases (STDs) among MSM have been found in several cities in China. Disturbing HIV prevalence rates from 1.0 to 5.0% among MSM have been reported in several urban cities [4]; higher than the overall prevalence (0.05%) for China. Without timely action, MSM could become the second most risky group for HIV infection following injection drug users in China.

In China, sociologists and public health workers have long been aware of the commercial sex workers serving MSM, who are called money boys. Beijing, for example, has thousands of male sex workers, working in bathhouses, bars and clubs or finding their own clients on the streets or via the internet [5,6]. It is shown in [5] that, even if money boys are normally managed by a so-called "Mommy", it is not uncommon for some of them to suffer physical violence and rape from clients [7]. In such circumstances, it is hardly realistic to hope that male sex workers will always use condoms. Migrant rural workers now also become a source of MSM [5,6]. China's fast-growing economy creates a lot of new jobs for migrant rural workers, who move frequently between big cities and their home town. A sizable proportion of these migrant workers have sex with men. Some of them also act as money boys to some old or not so popular gay men; in this situation, migrant workers normally only prefer insertive anal intercourse (AI) [6]. Thus, the population of money boys includes both professional money boys, and also a small number of rural workers. Some deterministic models have been proposed to understand the HIV epidemic in homosexual populations [8,9]. In [8], Valle et al looked at the impact of education, temporarily effective vaccines and therapies on the dynamics of HIV in homosexually active populations. Their study assumed that some individuals possess one or two mutant alleles (like D32 of CCR5) that prevent the successful invasion or replication of HIV, and the study examined separate or combined effects of therapies, education, vaccines and genetic resistance. Breban *et al*. [9] evaluated the potential impact of rectal microbicides for reducing HIV transmission in bathhouses. In addition, Tan & Kiang [10] proposed a state space model (Kalman filter model) for the HIV epidemic in homosexual populations stratified into sub-populations by their sexual activity levels.

**Figure 1:**
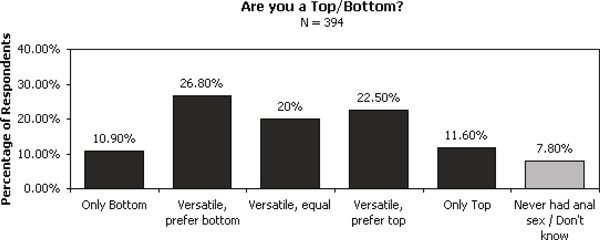
**Distribution of sex roles of MSM**. Sex roles should be thought of as a continuous spectrum that maps onto a continuous spectrum of physical preferences [11].

None of the aforementioned studies seems to have considered the sex-role preference, which is quite important to address the HIV spread in some MSM populations. In [11], Yee gave a summary of a study that tested a more elaborate categorization about the correlations between sex-role preference and physical preferences for partners among gay men. According to the sex roles of gay men, as Top or Bottom - preference for insertive AI and preference for receptive AI respectively - the study found that sex-role preference is indeed correlated with differences in physical preferences for a sexual partner among gay men. The categorization tested in this study includes 6 categories: Only Bottom; Versatile, but prefer Bottom; Versatile, equal; Versatile, but prefer Top; Only Top; Never had anal sex/Don't Know. All respondents in the study were volunteers recruited from http://www.gay.com chatrooms in January 2002. They were asked to participate in an online questionnaire. A total of 396 respondents completed the survey. Figure 1 gives the distribution of how respondents categorized themselves into the elaborate sex role categories above. The data of [11] suggests that sex roles should be thought of as a continuous spectrum that maps onto a continuous spectrum of physical preferences.

In our study here, we assume that HIV transmission takes place exclusively through AI (both receptive and insertive acts occur). We develop a mathematical model, based on the above categorization and the assumption that the viral transmission probability per anal sex act is different when transmission happens through receptive acts or insertive acts. Therefore, we divide MSM into three subgroups:

• Only Bottom, including gay and bisexual men who prefer receptive AI, and some money boys;

• Versatile, including all gay and bisexual men who are versatile in sex role, and some migrant rural workers;

• Only Top, including gay and bisexual men who prefer insertive AI, and some money boys (such as some migrant rural workers who earn subsidy income by having sex with gay men).

We use this model to examine the effect of highly active antiretroviral therapy on controlling the HIV spread in the MSM population. HAART has led to dramatic decrease in morbidity and mortality among individuals infected with HIV. However, HAART coverage remains suboptimal, even in the resource-rich areas of the world. Our model-based simulations therefore assume a small portion of MSM in China with HIV-1 will start to take HAART. Since HAART predictably decreases plasma HIV-1 RNA levels to below the levels of detection of currently available assays [12], we assume that individuals taking HAART are no longer infectious. An increase in adverse behaviour can result from the availability of interventions, the so-called disinhibition. Several early mathematical modeling studies raised the concern that any possible benefit of HAART on the spread of HIV could be readily offset by even modest increases in HIV risk behaviour [13]. However, our model shows that antiretroviral therapy for MSM in China will have both individual and public-health benefits even if risk behaviour increases. We also discuss the effect of vaccination for general MSM in order to compare different strategies.

## Methods

### Mathematical model

We develop an ordinary differential equation (ODE) model describing HIV infection among the MSM population according to individual's sex-role preference. We use *S*_*T*_, *S*_*B*_, *S*_*V *_to represent the susceptive MSM in the Only Top, the Only Bottom, and the Versatile category respectively. We use *I*_*T *_to represent the infected MSM in the Only Top category; *I*_*B *_and *I*_*V *_are defined similarly. Then we have the following ODE model:

where *N*_*T *_= *S*_*T *_+ *I*_*T*_, *N*_*V *_= *S*_*V *_+ *I*_*V *_and *N*_*B *_= *S*_*B *_+ *I*_*B *_denote the total population of the Only Top, the Versatile and the Only Bottom categories, respectively. Note that the Only Top category can have sex with the Only Bottom and the Versatile population. The Only Bottom category can have sex with the Only Top and the Versatile population. The Versatile category can have sex with all categories. In this model, we assume that susceptible and infected MSM can die at rates *d*_*M *_and *d*_*I *_respectively. Also, we assume new MSM are recruited into the appropriate susceptible compartment at rates *r*_*T*_, *r*_*B *_and *r*_*V *_respectively.

### The HIV transmission rate (*β*_*yx*_)

The key to quantify the transmission of HIV is the parameter *β*_*yx*_, the transmission rate that an individual from compartment *y *infects his partners from compartment *x*. We shall use the following formula to estimate the HIV transmission rate through either insertive or receptive AI:

The HIV transmission rate through anal sex in MSM depends on six quantities:

• the number of different AI sex partners per year, *n*_*x*_, for individuals from compartment *x*;

• the number of AI with each sex partner per year, *c*_*x*_, for individuals from compartment *x*;

• the viral transmission probability per anal sex act, *h*_*yx*_;

• the level of protection against HIV infection due to condom usage (if condoms are used, HIV transmission is decreased by a factor of (1 - *η*^*c*^*ρ*^*c*^), where *η*^*c *^is the condom efficacy and *ρ*^*c *^is the proportion of condom use);

• the proportion of infected MSM who know that they are infected, *α*_*y*_. This term denotes the effect of the 2008 HIV census in MSM population, where many men discovered they were HIV positive; *ν*_*y *_denotes the proportion of these infected MSM who begin to control their behaviour (such as condom use) to avoid the spreading of HIV, if they did not use condoms before they knew that they have been infected by HIV.

• other STIs increase both the rate of transmission and acquisition of HIV (the proportion with other STIs is assumed to be *ψ*^*s*^, with *μ*^*s *^being the multiplication factor for HIV);

### HAART and HIV vaccination in MSM

We assume that only 20% of MSM with HIV-1 start to take HAART each year in China, although some of the simulations below permit variable rates of HARRT treatment. To model the effects of HAART, we add one additional compartment to each of the infected groups. We assume that individuals taking HAART extend their lifespan by 5 years, so their annual death rate is 0.069. Since HAART predictably decreases plasma HIV-1 RNA levels to below the level of detection of currently available assays [12], we also assume that individuals taking HAART are no longer infectious. This reduction of plasma HIV-1 leads to an increase in adverse behaviour (disinhibition), and we model this behaviour change by reducing condom use between MSM from *ρ*^*c *^to zero (again, some of the simulations below allow for variable condom use rates). We also consider the effect of a potential vaccine, by adding one compartment for each of the uninfected groups. This vaccine has the property that vaccinated individuals may become infected, if the efficacy of the vaccine is less than 100%. In our baseline simulations, we assume that uninfected individuals are vaccinated at a rate of 20%, (the same as the HAART rate), and we explore vaccine efficacies of 30% and 70%. Equations for both the HAART model and the vaccination model can be found in Additional File 1.

### Parameters and initial values

Table 1, Table 2 and Table 3 describe values of each of the biological and behavioural parameters, and Table 4 gives initial values. Some parameter values are calculated from the cohort study of China in 2008, which is on "high-risk behaviours and HIV/syphilis prevalence among men who have sex with men in Beijing". We also give the standard error for these parameter values. For example, the mean value of *n*_*V *_(the number of AI sex partner per year of Versatile MSM) is 12.4 and the standard error is 12.2. The mean value of *c*_*V *_(the number of AI with each sex partner per year of the Versatile) is 4.5 and the standard error is 4.4. The large variation in our original data - for example, the minimum value of *n*_*V *_is 1 but the maximum value is 120 - results in the large standard errors of the parameters.

**Table 1:** Parameters.

Para	Description	Value	Source
*r*_*T*_	source rate of Only-Top MSM	96667	estimate
*r*_*V*_	source rate of Versatile MSM	603950	estimate
*r*_*B*_	source rate of Only-Bottom MSM	70817	estimate
*d*_*M*_	death rate of susceptible MSM	0.022	estimate
*d*_*I*_	death rate of infected MSM	0.105	estimate
*n*_*T*_	number of AI sex partners per year of Only-Top MSM	11.5	[16]
*n*_*V*_	number of AI sex partners per year of Versatile MSM	12.4	[16]
*n*_*B*_	number of AI sex partner per year of Only-Bottom MSM	13.6	[16]
*c*_*T*_	number of AI acts with each sex partners per year of Only-Top MSM	4.4	[16]
*c*_*V*_	number of AI acts with each sex partners per year of Versatile MSM	4.5	[16]
*c*_*B*_	number of AI acts with each sex partners per year of Only-Bottom MSM	4.2	[16]
*h*_*TB*_	transmissibility of HIV from Top to Bottom	0.01	[9,20]
*h*_*VB*_	transmissibility of HIV from Versatile to Bottom	0.01	[9,20]
*h*_*TV*_	transmissibility of HIV from Top to Versatile	0.01	[9,20]
*h*_*BT*_	transmissibility of HIV from Bottom to Top	0.005	[9,20]
*h*_*VT*_	transmissibility of HIV from Versatile to Top	0.005	[9,20]
*h*_*BV*_	transmissibility of HIV from Bottom to Versatile	0.005	[9,20]
*h*_*VV*_	transmissibility of HIV from Versatile to Versatile	0.0075	estimate
α_*y*_	proportion of infected MSM who know they are infected	0.15	[16]
*η*^*c*^	condom efficacy	0.9	[16]
*ρ*^*c*^	rate of condom use	0.3	[16]
*ψ*^*s*^	proportion with STI	0.158	[16]
*μ*^*s*^	multiplication factor of STI for HIV	2.89	[21]
*ν*_*y*_	proportion of infected MSM who begin to control their behaviour	0.5	[16]

Note 1: The mean values and standard errors of parameters *n*_*T*_, *n*_*V*_, *n*_*B*_, *c*_*T*_, *c*_*V *_and *c*_*B *_are

respectively.

Note 2: We estimate that the average life span of HIV infected individual is 9.5 years, so *d*_*I *_= 0.105. Also, we suppose young MSM begin homosexual sex behavior at 20 years and quit at 65 years, so *d*_*M *_= 0.022.

**Table 2:** Parameters and *R*_0_.

Parameters	Case 1	Case 2	Case 3
*h*_*TB*_, *h*_*VB*_, *h*_*TV*_	0.01	0.001	0.001
*h*_*BT*_, *h*_*VT*_, *h*_*BV*_	0.005	0.0005	0.0005
*h*_*VV*_	0.0075	0.00075	0.00075
*ρ*^*c*^	0.3	0.6	0.6
*d*_*I*_	0.105	0.105	0.026

*R*_0_	3.9296	0.2528	1.0212

**Table 3:** Infection rates of Table 1.

Compartment	Description	Value
*β*_*TB*_	rate of infection by *I*_*T *_of *S*_*B*_	0.5786
*β*_*TV*_	rate of infection by *I*_*T *_of *S*_*V*_	0.5653
*β*_*BT*_	rate of infection by *I*_*B *_of *S*_*T*_	0.2563
*β*_*VB*_	rate of infection by *I*_*V *_of *S*_*B*_	0.5786
*β*_*VT*_	rate of infection by *I*_*V *_of *S*_*T*_	0.2563
*β*_*BV*_	rate of infection by *I*_*B *_of *S*_*V*_	0.2826
*β*_*VV*_	rate of infection by *I*_*V *_of *S*_*V*_	0.4239

For the initial values, we have the following. The cohort study in China in 2008 [16] estimated that the prevalence of HIV in MSM in China was about 1.5% by the end of 2008 [16]. The total population of China is about 1.3029 × 10^9^, and 50% of them are male. We suppose 3% of men in China have sex with men, 91.5% of whom experience AI, distributed as in Figure 1: 10.9% are Only-Top, 69.3% are Versatile and 11.6% are Only-Bottom. Then we have 1.8214 × 10^7 ^MSM experiencing AI, which is in the range estimated by [3]: the total number of gays in China is between 1.8 × 10^7 ^and 2.4 × 10^7^. We also estimate that the number of professional money boys is about 10,000. According to the newest report, the estimated number of rural workers was 2.2542 × 10^8 ^at the end of 2008 [14], 56% of whom are male. The study [5] estimated that about 10% of rural workers have homosexual sex behaviours to various extent, in which 20% experience AI. We suppose that these AI-rural workers follow the distribution of 10.9% Only-Top (1% of them are money boys), 69.3% Versatile and 11.6% Only-Bottom. In summary, we estimate that the total number of MSM in China is about 2.0745 × 10^7^. The initial values for each compartment can be found in Table 4.

**Table 4:** Initial Conditions.

Compartment	Description	Initial Value
*S*_*T*_	susceptible Only-Top MSM	2.4251 × 10^6^
*S*_*V*_	susceptible Versatile MSM	1.5418 × 10^7^
*S*_*B*_	susceptible Only-Bottom MSM	2.5908 × 10^6^
*I*_*T*_	infected Only-Top MSM	3.6931 × 10^4^
*I*_*V*_	infected Versatile MSM	2.3480 × 10^5^
*I*_*B*_	infected Only-Bottom MSM	3.9455 × 10^4^

## Results and discussion

### Model analysis: the basic reproductive ratio, *R*_0_

Following the next-generation operator method of [15], we linearize the second, the fourth and the sixth equations of our model around the disease-free state and look for conditions that guarantee the growth of the three infected classes, *I*_*T*_, *I*_*V *_and *I*_*B*_.

For simplicity, we write *β*_*VT *_instead of . Other parameters labelled *β*_*xy *_have similar meanings. The Jacobian matrix of the second, the forth and the sixth equations of our model at the disease-free state can be rewritten in the following form:

Where

and

*R*_0 _is the spectral radius of the next-generation matrix. Therefore, to find *R*_0 _we must find the largest eigenvalue of .

So

that is

Then the characteristic equation of  is

Where

Let

and . Then

The reproductive number, *R*_0_, is the number of secondary cases produced by a typical infected individual during his entire period of infectiousness in a demographically steady susceptible population. Calculating this number for our model is critical to determine whether HIV can invade the MSM population and/or stabilize in the population. We can show that when *R*_0 _< 1, HIV will not be sustained in this MSM population; otherwise, the infection will approach an endemic equilibrium of constant incidence and prevalence.

### Persistence of HIV infection

By Thieme's persistence theory, we prove that the system is persistent of HIV infection when *R*_0 _> 1; i.e., when *R*_0 _> 1, HIV will spread in the MSM population so long as one infected MSM is introduced in this population, regardless of whether he is an Only-Top, a Versatile or an Only-Bottom. We also get the existence of disease equilibrium (or equilibria) from the persistence of HIV infection. The proof is in Additional File 1.

### Numerical simulations

#### Outcomes without any intervention

**Figure 2:**
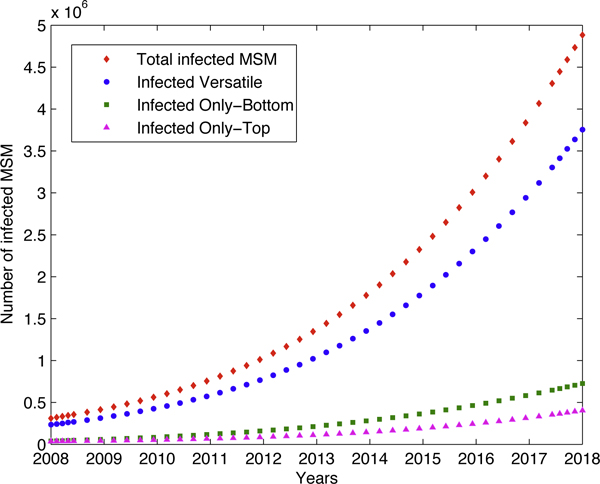
**Dynamics of all sub-populations in the absence of intervention**. Parameters used were as in Table 1. Initial conditions were as in Table 4. With these parameters, we have *R*_0_=3.9296 and hence the disease-free equilibrium is unstable and the disease is uniformly persistent. By the year 2018 the total number of newly infected MSM in China will be close to 4.88 x 10^6^. That is, prevalence among MSM will reach over 21.7% by the year 2018 and the Versatile MSM alone will reach a prevalence rate of over 16.6%.

We start with the baseline analysis where we assume there is no intervention measure. Parameter values are chosen as in Table 1, estimated from the situation of 2008 in China [16]. We obtained that *R*_0 _= 3.9296. In other words, one HIV-infected MSM can infect almost 4 other HIV-susceptible individuals in the same population before he dies. Since *R*_0 _> 1, epidemic will be firmly established, and our simulation (Figure 2) predicts that, by the year 2018, the total number of newly infected MSM in China will be close to 4.88 × 10^6^, and HIV prevalence among MSM will reach over 21.7%. The infected Versatile MSM population will have a HIV prevalence rate of over 16.6%. Note that the infected Versatile MSM increases much faster than the other two sub-populations (Figure 2). It shows the higher risk of this subpopulation if no intervention measure is implemented.

**Figure 3:**
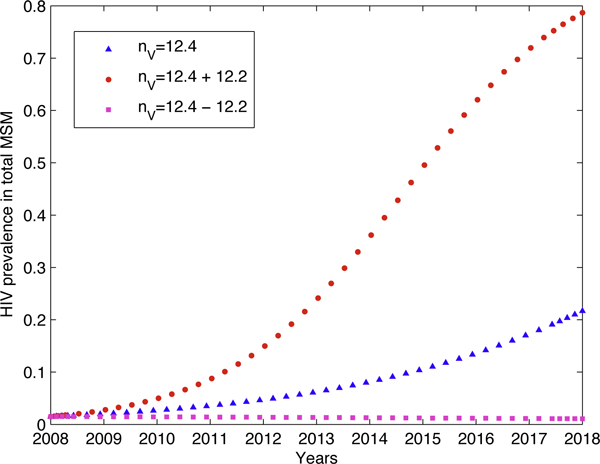
**The outcome varies with errors in the parameter estimates**. The effect of different *n*_*V *_(the number of AI sex partner per year of Versatile MSM) on the outcome of ten years' prevalence prediction. The mean value of *n*_*V *_= 12.4 and its standard error is 12.2. *R*_0 _= 7.1064 for *n*_*V *_= 24.6, which is almost double of *R*_0 _= 3.9296 of *n*_*V *_= 12.4.

As far as the 10-year HIV prevalence prediction above is concerned, the outcomes are robust to most of the parameters in Table 1, except some special ones. Simulations show that the number of AI sex partners per year of Versatile MSM (*n*_*V*_) and the number of AI events with each sex partner per year for Versatile MSM (*c*_*V*_) are the two parameters which influence the final outcome most, and the condom use rate (*ρ*^*c*^) the least. Figure 3 shows the effect of different values of *n*_*V *_on the outcome of ten years' prevalence prediction, which shows the critical role played by the number of sex partners.

#### Outcomes of HAART and vaccination

**Figure 4:**
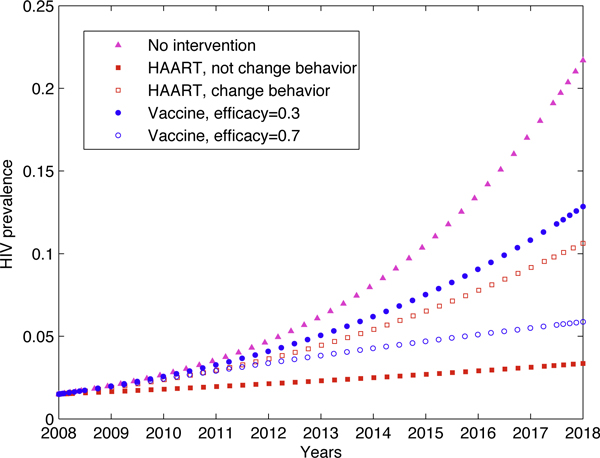
**Comparison of dynamics for HIV-infected MSM**. Comparison of dynamics for HIV infected MSM: no intervention, HAART and vaccination. All parameters used were the same as in Figure 2, except the condom use rate *ρ*^*c *^was either 0.30 or 0. Additionally, for the HAART strategy, individuals taking HAART extend their lifespan by 5 years. So their death rate *d*_*A *_is 0.069. For the vaccination strategy, uninfected individuals were vaccinated at a rate of 20%, and the effect of the vaccine is 30% and 70% respectively. For these parameters, we have  = 1.3528 for *ρ*^*c *^= 0.30 (i.e., MSM do not change behaviour),  = 1.8138 for *ρ*^*c *^= 0 (i.e., MSM change behaviour),  = 2.8676 for 30% efficacy and  = 1.4515 for 70% efficacy. Whether MSM population change their behaviour or not after receiving HAART (in our setting, this means that the condom use rate remains at *ρ*^*c *^= 0.30 or decreases to zero), prevalence can be greatly reduced.

The effects of HAART and vaccination are illustrated in Figure 4. Suppose the HAART rate in MSM is 20%. If MSM population will not change their behaviour after receiving HAART (in our setting, this means that the condom using rate remains as *ρ*^*c *^= 0.3), then *R*_0 _= 1.3528 and the prevalence can be greatly reduced (the solid-square curve). The empty-square curve shows the effect of HAART with behaviour change (disinhibition occurs, with the condom using rate *ρ*^*c *^= 0). *R*_0 _= 1.8138 at this situation, which is still smaller than that without any intervention.

Suppose the vaccination rate in MSM is also 20%. Then the effects of a potential vaccine with efficacy of 30% and 70% are shown as the solid-ring curve and the empty-ring curve, respectively. *R*_0 _= 2.8676 and *R*_0 _= 1.4514 for each situation. It shows that the effect of a potential vaccine appears worse than that of HAART, even if the vaccine efficacy is as high as 70%. Simulation shows that even if disinhibition occurs, the effect of HAART is still much better than that of no intervention. This result is remarkably different from those in [13], but agrees with those in [17].

**Figure 5:**
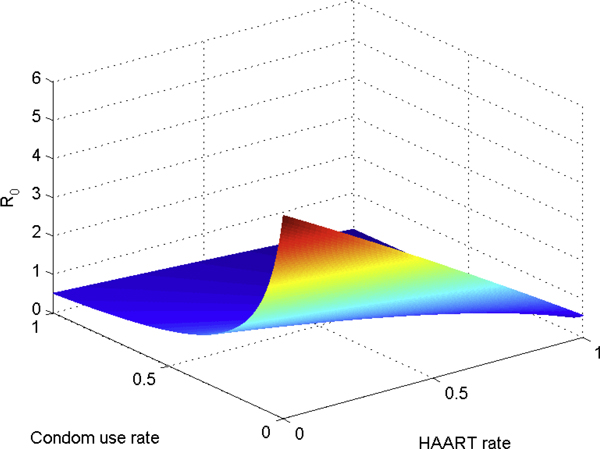
***R*_0 _as a function of the HAART and condom rates**. *R*_0 _as a function of the HAART and condom rates. All other parameters were the same as in Figure 4. This figure illustrates the feasibility of controlling the HIV epidemic within the MSM population via a combination of condom use and HAART program.

**Figure 6:**
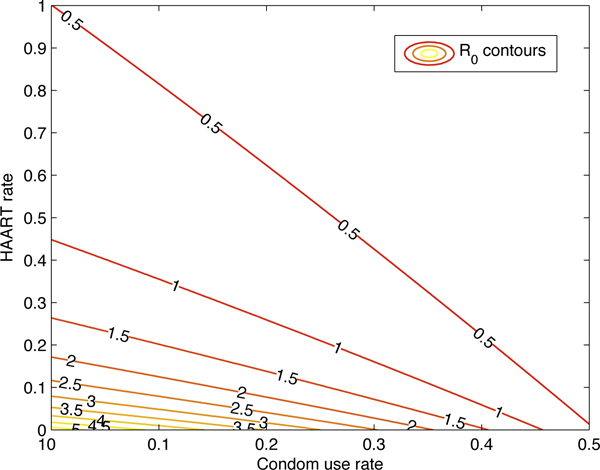
**Contour plots of *R*_0 _as HAART and condom rates vary**. Contour plots of *R*_0 _as HAART and condom rates vary.

**Figure 7:**
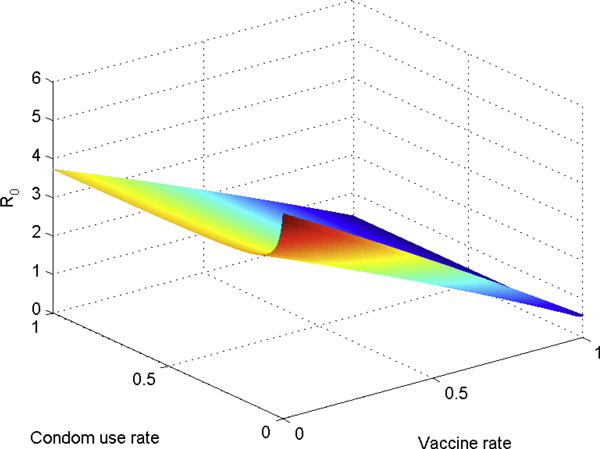
***R*_0 _as a function of the vaccine and condom rates**. *R*_0 _as a function of the vaccine and condom rates. This figure illustrates the feasibility of controlling the HIV epidemic within the MSM population via a combination of condom use and vaccine program.

**Figure 8:**
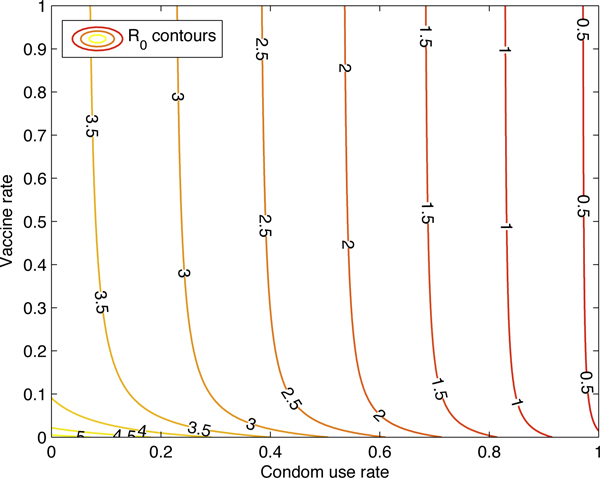
**Contour plots of *R*_0 _as vaccine and condom rates vary**. Contour plots of *R*_0 _as vaccine and condom rates vary.

Figure 5 shows the graph of *R*_0 _as a function of the condom rate and the HAART rate, while Figure 6 plots a few contours of *R*_0_. Figure 7 gives the graph of *R*_0 _as a function of the vaccination rate, while Figure 8 plots a few contours of *R*_0_. These illustrate the feasibility of controlling the HIV epidemic within the MSM population via a combination of condom use and HAART or a vaccination program.

#### Reproduction number and lifespan: power law

**Figure 9:**
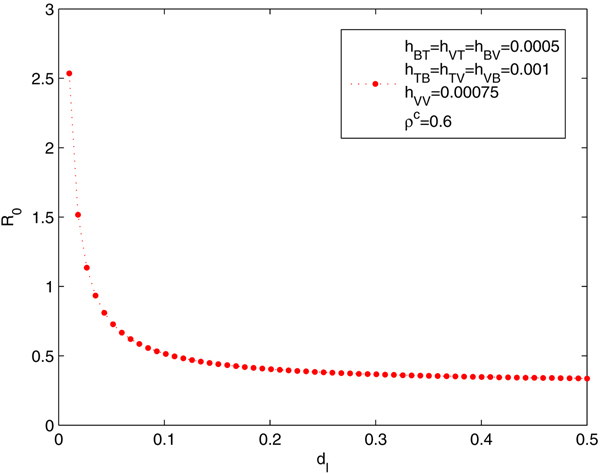
**The relation between *R*_0 _and *d*_*I *_ follows a power law**. The relation between *R*_0 _and *d*_*I*_, power law. Parameters used were as in Case 3 of Table 2. *R*_0 _is a decreasing function of the death rate *d*_*I*_, following a power law at least asymptotically.

It was reported that certain medicines, especially some traditional Chinese medicines [18,19], which are reasonably cheap and easily available to HIV-infected individuals, can decrease the viral load and extend the lifespan of HIV-infected individuals. Here we design a simulation to simulate the impact of such medicines: we assume the transmission probability per anal sex is 10 times less than without the medicine, and the HIV-infected individuals have the reduced death rate *d*_*I *_= 0.026. Even under the assumption that MSM are more cautious in sexual behaviour (so that their condom using rate is doubled), we noticed that an endemic is maintained in the population since the basic production ratio *R*_0 _= 1.0212 > 1 (Case 3 in Table 2, in comparison with those in Case 2). One of the reasons for this sustained infection is that the ratio *R*_0 _is an decreasing function of the death rate *d*_*I*_, following a power law at least asymptotically (See Figure 9).

## Conclusion

We developed a mathematical model using a sex-role-preference framework to predict HIV infection in the MSM population. An analytic expression of the basic reproduction ratio *R*_0 _was obtained using model parameters, and we estimated the current *R*_0 _as 3.9296.

Our simulations suggest that both antiretroviral therapy and a potential vacce are powerful interventions, even if disinhibition is considered. Our simulations also suggest that having protected sexual behaviour has limited effect on controlling an epidemic in the MSM population, and medicine which can reduce the transmission and extend the lifespan of the infected has a complex impact.

There are three points that we should pay attention to. First, we suppose that most of these professional money boys are being in the Bottom Only category. This is from the investigation of China's current situation. Maybe there are some of them being in the other two categories. But since the proportions are small, the effect should be also very limit to the final outcomes. Second, considering the large variation of the data of different sexual partners for each MSM, which is possible to obey a power-law distribution, maybe the complex network (such as the Scale-Free network) is a more suitable method to model the spreading of HIV in MSM. This is also what we want to try in our next work. Third, many MSM in China, whether occasionally or frequently having sex with men, do not necessarily regard themselves as homosexual or bisexual. They are very often married. Even if they are not, they may have sex with women as well. This applies particularly to those societies wherein marriage is strongly promoted by the society and the family. This is largely true for rural workers, most of whom are married. Thus, as a bridging population, infected MSM transmit the infection to their heterosexual partners and thereafter to the general community.

## Competing interests

The authors declare that they have no competing interests.

## Authors' contributions

JL and JW are responsible for the model building and theory analysis. LC is responsible for simulations. YR conducted data collection and analysis. YS is responsible for designing the study, identifying the key indicators, and providing scientific discussion and application of them to the epidemic and public health efforts.

## Supplementary Material

Additional file 1 Click here for file
