# Supplementary material for: A sex-role-preference model for HIV transmission among men who have sex with men in China
Source: BMC Public Health. 2009 Nov 18;9(Suppl 1):S10. doi: 10.1186/1471-2458-9-S1-S10 (PMC2779498; doi:10.1186/1471-2458-9-S1-S10)
Supplement: Additional file 1 [file 1471-2458-9-S1-S10-S1.pdf]

Additional File 1
Equations of HAART and vaccination strategy
HAART strategy

Let A_T, A_V and A_B denote the Only-Top, Versatile and Only-Bottom MSM population respectively that were treated. Then we have the following equations for the HAART strategy:

$$
\begin{cases}
\frac{dS_T}{dt} &= r_T - S_T\left(\frac{\beta_{I_B S_T} I_B + \beta_{I_V S_T} I_V}{\check{N}_B + \check{N}_V}\right) - d_M S_T \\
\frac{dI_T}{dt} &= S_T\left(\frac{\beta_{I_B S_T} I_B + \beta_{I_V S_T} I_V}{\check{N}_B + \check{N}_V}\right) - d_I I_T - aI_T \\
\frac{dA_T}{dt} &= aI_T - d_A A_T \\
\frac{dS_V}{dt} &= r_V - S_V\left(\frac{\beta_{I_T S_V} I_T + \beta_{I_B S_V} I_B + \beta_{I_V S_V} I_V}{\check{N}_T + \check{N}_V + \check{N}_B}\right) - d_M S_V \\
\frac{dI_V}{dt} &= S_V\left(\frac{\beta_{I_T S_V} I_T + \beta_{I_B S_V} I_B + \beta_{I_V S_V} I_V}{\check{N}_T + \check{N}_V + \check{N}_B}\right) - d_I I_V - aI_V \\
\frac{dA_V}{dt} &= aI_V - d_A A_V \\
\frac{dS_B}{dt} &= r_B - S_B\left(\frac{\beta_{I_T S_B} I_T + \beta_{I_V S_B} I_V}{\check{N}_T + \check{N}_V}\right) - d_M S_B \\
\frac{dI_B}{dt} &= S_B\left(\frac{\beta_{I_T S_B} I_T + \beta_{I_V S_B} I_V}{\check{N}_T + \check{N}_V}\right) - d_I I_B - aI_B \\
\frac{dA_B}{dt} &= aI_B - d_A A_B,
\end{cases}
$$

where $\check{N}_i = S_i + I_i + A_i$, $i = T, V, B$.

Vaccination strategy

Let V_T, V_V and V_B denote the Only-Top, Versatile and Only-Bottom MSM population respectively that were vaccinated. Then we have the following equations for the vaccination strategy:

$$
\begin{cases}
\frac{dS_T}{dt} &= r_T - S_T\left(\frac{\beta_{I_B S_T} I_B + \beta_{I_V S_T} I_V}{\tilde{N}_B + \tilde{N}_V}\right) - d_M S_T - v S_T \\
\frac{dI_T}{dt} &= S_T\left(\frac{\beta_{I_B S_T} I_B + \beta_{I_V S_T} I_V}{\tilde{N}_B + \tilde{N}_V}\right) + V_T\left(\frac{\beta_{I_B S_T} I_B + \beta_{I_V S_T} I_V}{\tilde{N}_B + \tilde{N}_V}\right)(1-\epsilon) - d_I I_T \\
\frac{dV_T}{dt} &= v S_T - V_T\left(\frac{\beta_{I_B S_T} I_B + \beta_{I_V S_T} I_V}{\tilde{N}_B + \tilde{N}_V}\right)(1-\epsilon) - d_M V_T \\
\frac{dS_V}{dt} &= r_V - S_V\left(\frac{\beta_{I_T S_V} I_T + \beta_{I_B S_V} I_B + \beta_{I_V S_V} I_V}{\tilde{N}_T + \tilde{N}_V + \tilde{N}_B}\right) - d_M S_V - v S_V \\
\frac{dI_V}{dt} &= S_V\left(\frac{\beta_{I_T S_V} I_T + \beta_{I_B S_V} I_B + \beta_{I_V S_V} I_V}{\tilde{N}_T + \tilde{N}_V + \tilde{N}_B}\right) \\
& \quad + V_V\left(\frac{\beta_{I_T S_V} I_T + \beta_{I_B S_V} I_B + \beta_{I_V S_V} I_V}{\tilde{N}_T + \tilde{N}_V + \tilde{N}_B}\right)(1-\epsilon) - d_I I_V \\
\frac{dV_V}{dt} &= v S_V - V_V\left(\frac{\beta_{I_T S_V} I_T + \beta_{I_B S_V} I_B + \beta_{I_V S_V} I_V}{\tilde{N}_T + \tilde{N}_V + \tilde{N}_B}\right)(1-\epsilon) - d_M V_V \\
\frac{dS_B}{dt} &= r_B - S_B\left(\frac{\beta_{I_T S_B} I_T + \beta_{I_V S_B} I_V}{\tilde{N}_T + \tilde{N}_V}\right) - d_M S_B - v S_B \\
\frac{dI_B}{dt} &= S_B\left(\frac{\beta_{I_T S_B} I_T + \beta_{I_V S_B} I_V}{\tilde{N}_T + \tilde{N}_V}\right) + V_B\left(\frac{\beta_{I_T S_B} I_T + \beta_{I_V S_B} I_V}{\tilde{N}_T + \tilde{N}_V}\right)(1-\epsilon) - d_I I_B \\
\frac{dV_B}{dt} &= v S_B - V_B\left(\frac{\beta_{I_T S_B} I_T + \beta_{I_V S_B} I_V}{\tilde{N}_T + \tilde{N}_V}\right)(1-\epsilon) - d_M V_B
\end{cases}
$$

where $\tilde{N}_i = S_i + I_i + V_i$, $i = T, V, B$ and ϵ is the vaccine efficacy, satisfying $0 \le \epsilon \le 1$.

The persistence of HIV infection

To study the persistence of infection, we discuss the equivalent system of our model as follows:

$$\begin{cases} \dfrac{dI_T}{dt} &= (N_T - I_T)\left(\dfrac{\beta_{I_B S_T} I_B + \beta_{I_V S_T} I_V}{N_B + N_V}\right) - d_I I_T \\ \dfrac{dN_T}{dt} &= r_T - d_M N_T - (d_I - d_M) I_T \\ \dfrac{dI_V}{dt} &= (N_V - I_V)\left(\dfrac{\beta_{I_T S_V} I_T + \beta_{I_B S_V} I_B + \beta_{I_V S_V} I_V}{N_T + N_V + N_B}\right) - d_I I_V \\ \dfrac{dN_V}{dt} &= r_V - d_M N_V - (d_I - d_M) I_V \\ \dfrac{dI_B}{dt} &= (N_B - I_B)\left(\dfrac{\beta_{I_T S_B} I_T + \beta_{I_V S_B} I_V}{N_T + N_V}\right) - d_I I_B \\ \dfrac{dN_B}{dt} &= r_B - d_M N_B - (d_I - d_M) I_B. \end{cases} \tag{1}$$

The positive invariant domain of system (1) is

$$D = \{(I_i, N_i) : 0 \le I_i \le N_i, 0 \le N_i \le K_i\}, \quad i = T, V, B$$

and the disease-free equilibrium is

$$E_0 = (I_T^0, N_T^0, I_V^0, N_V^0, I_B^0, N_B^0) = \left(0,\ \frac{r_T}{d_M},\ 0,\ \frac{r_V}{d_M},\ 0,\ \frac{r_B}{d_M}\right).$$

First, we introduce some basic definitions and a lemma that will be useful for our discussion. More definitions and results about persistence can be found in [1].

Let X be a locally compact metric space, with metric d. Let X be the disjoint union of two sets X_1 and X_2 such that X_2 is compact. Let Φ be a continuous semi-flow on X_1. An invariant subset M of X is said to be isolated if M is the maximal invariant set in some neighborhood of itself. Let A and B be two isolated invariant sets. A is chained to B ($A \to B$) if there is a full orbit through x which is not either in A or in B, such that $\omega(x) \subset B, \alpha(x) \subset A$. Moreover, a finite sequence $\{M_1, M_2, ..., M_k\}$ of invariant sets is also called a chain if $M_1 \to M_2 \to ... \to M_k$. The chain is called cyclic if $M_k = M_1$. Otherwise, it is called acyclic.

Lemma 1 (Proposition 4.3 in [1]). *Let X be locally compact, X_2 be compact in X and X_1 be forward invariant under the continuous semiflow Φ on X. Let x_n be a sequence of elements in X_1 satisfying*

$$\lim_{t\to\infty} \sup d(\Phi_t(x_n), X_2) \to 0, \qquad n \to \infty.$$

Let $M = \cup_{k=1}^m M_k$ be an isolated covering of Ω_2 such that $\omega(x_n)$ not belong to M_k for all n, k. Then M is cyclic.

Then for the persistence of infection, we have the following Theorem.

Theorem 1. *When $R_0 > 1$, system (1) is uniformly persistent of infection, i.e., there exists $\varepsilon > 0$ for system (1), such that*

$$\lim_{t\to\infty} \inf \min\{I_T(t), I_V(t), I_B(t)\} > \varepsilon,$$

for any solution $x(t)$ with $N_T(0) > 0, N_V(0) > 0, N_B(0) > 0$ and any one of the three initial conditions holds: $I_T(0) > 0, I_V(0) > 0$ or $I_B(0) > 0$.

Proof. First we calculate the Jacobian matrix of system (1) at E_0. It is more convenient to change the order of coordinates to $I_T, I_V, I_B, N_T, N_V, N_B$ to study the Jacobian matrix.

The Jacobian matrix can be written as follows

$$J|_{E_0} = \begin{bmatrix} J_{LT} & 0 \\ J_{LB} & J_{RB} \end{bmatrix},$$

where

$$J_{LT} = \begin{bmatrix} -d_I & \dfrac{r_T\beta_{VT}}{r_B + r_V} & \dfrac{r_T\beta_{BT}}{r_B + r_V} \\ \dfrac{r_V\beta_{TV}}{r_T + r_V + r_B} & \dfrac{r_V\beta_{VV}}{r_T + r_V + r_B} - d_I & \dfrac{r_V\beta_{BV}}{r_T + r_V + r_B} \\ \dfrac{r_B\beta_{TB}}{r_T + r_V} & \dfrac{r_B\beta_{VB}}{r_T + r_V} & -d_I \end{bmatrix},$$

$$J_{LB} = \begin{bmatrix} -(d_I - d_M) & 0 & 0 \\ 0 & -(d_I - d_M) & 0 \\ 0 & 0 & -(d_I - d_M) \end{bmatrix}$$

and

$$J_{RB} = \begin{bmatrix} -d_M & 0 & 0 \\ 0 & -d_M & 0 \\ 0 & 0 & -d_M \end{bmatrix}.$$

Define

$$\begin{aligned} D_2 &= \{(I_T, N_T, I_V, N_V, I_B, N_B) | I_T = 0, \, or \, I_V = 0, \, or \, I_B = 0, 0 \le N_i \le K_i\}, \\ D_1 &= D \backslash D_2, \\ \tilde{D}_1 &= \{(I_T, N_T, I_V, N_V, I_B, N_B) | 0 < I_i < N_i, 0 < N_i \le K_i\} \end{aligned}$$

where $i = T, V, B$ respectively, and D_1 and $\tilde{D}_1$ are forward invariant.

Let $x^0 = (I_T(0), N_T(0), I_V(0), N_V(0), I_B(0), N_B(0))$. From system (1) and the assumptions ($N_i(0) > 0$ and at least any one of $I_i(0) > 0$, $i = T, V, B$ holds), it is easy to get that $\Phi_t(x^0) \in \tilde{D}_1$ for all $t > 0$. So we can then assume $x_0 \in \tilde{D}_1$.

Define $\Omega_2 = \cup_{x \in D_2} \omega(x)$. It is easy to see that $\Omega_2 = \{E_0\}$. Then we will prove the following:

1. $\{E_0\}$ is a weak repeller for $\tilde{D}_1$;

2. D_2 is a uniform weak repeller for $\tilde{D}_1$;

3. D_2 is a uniform strong repeller for $\tilde{D}_1$.

First, let's prove that $\{E_0\}$ is a weak repeller for $\tilde{D}_1$. Suppose $x(t)(= \Phi_t(x^0))$ stays in a small neighborhood of E_0. Then we have two cases:

1. if $I_T(0) = I_V(0) = I_B(0) = 0$, then $I_T(t) = I_V(t) = I_B(t) \equiv 0$. System (1) shows that $(N_T(t), N_V(t), N_B(t))$ goes far away from E_0 as $t \to -\infty$.

2. if $I_T(0) > 0$, or $I_V(0) > 0$, or $I_B(0) > 0$ holds, then $I_T(t) > 0, I_V(t) > 0$ and $I_B(t) > 0$ for all $t > 0$. When $x(t)$ stays very close to E_0, by system (1), we know that there exists some $\delta > 0$ which is related to the size of the neighborhood of E_0, such that

$$\frac{dX}{dt} > J_\delta X, \tag{2}$$

where

$$J_\delta = \begin{bmatrix} J_{LT}^{11} - \delta & J_{LT}^{12} - \delta & J_{LT}^{13} - \delta \\ J_{LT}^{21} - \delta & J_{LT}^{22} - \delta & J_{LT}^{23} - \delta \\ J_{LT}^{31} - \delta & J_{LT}^{32} - \delta & J_{LT}^{33} - \delta \end{bmatrix}.$$

$J_{LT}^{ij}(i, j = 1, 2, 3)$ are the entries of the top-left matrix J_{LT} of the matrix $J|_{E_0}$. Since $R_0 > 1$, then by choosing δ small enough, J_δ has positive non-diagonal elements and its largest eigenvalue is positive. Hence, the solutions of the linear quasi-monotonic system

$$\frac{dY}{dt} = J_\delta Y,$$

where

$$Y = \begin{bmatrix} y_1, & y_2, & y_3 \end{bmatrix}^T,$$

with $y_1(0) > 0, y_2(0) > 0, y_3(0) > 0$ are exponentially increasing as $t \to \infty$. By the comparison principle, $(I_T(t), I_V(t), I_B(t))$ goes far away from $(0, 0, 0)$.

From the above two cases, $\{E_0\}$ is isolated in D and it cannot be chained to itself in D_2, i.e., $\{E_0\}$ is an acyclic covering for Ω_2. From the proof of case 2 we know that $\{E_0\}$ is a weak repeller for $\tilde{D}_1$.

Second, let's prove that D_2 is a uniform weak repeller for $\tilde{D}_1$. If D_2 is not a uniform weak repeller for $\tilde{D}_1$, then we can find a sequence

$$x_n = (I_{Tn}, N_{Tn}, I_{Vn}, N_{Vn}, I_{Bn}, N_{Bn}) \in \tilde{D}_1 \subset D_1,$$

satisfying

$$\lim_{t\to\infty} \sup d(\Phi_t(x_n), D_2) \to 0, \quad n \to \infty.$$

As $\{E_0\}$ is a weak repeller for $\tilde{D}_1$, we have $\omega(x_n)$ not belong to $\{E_0\}$ for all n. Using Lemma 1, we get that $\{E_0\}$ should be cyclic, which is contrary to our discussion above. So D_2 is a uniform weak repeller for $\tilde{D}_1$; i.e., there exists $\tilde{\varepsilon} > 0$ such that

$$\lim_{t\to\infty} \sup \min\{I_T(t), I_V(t), I_B(t)\} > \tilde{\varepsilon} \tag{3}$$

for any solution $x(t)$ with $I_i(0) > 0, i = T, V, B$.

Finally, let's prove that D_2 is a uniform strong repeller for $\tilde{D}_1$. Suppose that D_2 is not a uniform strong repeller for $\tilde{D}_1$. Then there exist sequences

$$x_j^0 = (I_T^j(0), N_T^j(0), I_V^j(0), N_V^j(0), I_B^j(0), N_B^j(0)) \in \tilde{D}_1$$

and $0 < \varepsilon_j < \tilde{\varepsilon}$, such that

$$\lim_{t\to\infty} \inf \min\{I_T^j(t), I_V^j(t), I_B^j(t)\} < \varepsilon_j \quad \text{for} \quad j = 1, 2, ... \tag{4}$$

Here, $\lim_{t\to\infty} \varepsilon_j = 0$ and $(I_T^j(t), N_T^j(t), I_V^j(t), N_V^j(t), I_B^j(t), N_B^j(t))$ are the solutions of system (1) with initial values $x_j^0 \in \tilde{D}_1$.

From (3) and (4) we can find sequences $0 < r_j < s_j < t_j$ with $\lim_{j\to\infty} r_j = \infty$ such that

$$\lim_{j\to\infty} \min\{I_T^j(s_j), I_V^j(s_j), I_B^j(s_j)\} = 0, \tag{5}$$

$$\min\{I_T^j(r_j), I_V^j(r_j), I_B^j(r_j)\} = \min\{I_T^j(t_j), I_V^j(t_j), I_B^j(t_j)\} = \tilde{\varepsilon} \tag{6}$$

and

$$\min\{I_T^j(r_j), I_V^j(r_j), I_B^j(r_j)\} \leq \tilde{\varepsilon} \quad \text{for} \;\; r_j \leq t \leq t_j. \tag{7}$$

Now, for sequence $(I_T^j(r_j), N_T^j(r_j), I_V^j(r_j), N_V^j(r_j), I_B^j(r_j), N_B^j(r_j))$, which is convergent, from (6) we say it converges to

$$(I_T^*(0), N_T^*(0), I_V^*(0), N_V^*(0), I_B^*(0), N_B^*(0)) = x^*(0) \in \tilde{D}_1$$

when $j \to \infty$.

Now we prove that $t_j - r_j$ is unbounded when $j \to \infty$. Suppose it is not true. Then, after taking a subsequence, $s_j - r_j$ converge to s^* when $j \to \infty$.

Let $x^*(t)$ denote the solution of system (1) with initial value $x^*(0) \in \tilde{D}_1$. Then, according to the basic properties of flow and the fact that $\tilde{D}_1$ is invariant, we have

$$+s^*), \lim_{j\to\infty}(I_i^j(r_j + s^*), N_i^j(r_j + s^*)) = x^*(s^*) \in \tilde{D}_1, \quad i = T, V, B. \tag{8}$$

From (5), we have

$$\lim_{j\to\infty}(I_T^j(s_j), N_T^j(s_j), I_V^j(s_j), N_V^j(s_j), I_B^j(s_j), N_B^j(s_j)) = x^*(s^*) \in D_2, \tag{9}$$

which is a contradiction. So we say $t_j - r_j$ is unbounded when $j \to \infty$.

Now let $x^*(0) \in \tilde{D}_1$. Then, from (3), we have

$$\lim_{t\to\infty} \sup \min\{I_T^*(t), I_V^*(t), I_B^*(t)\} > \tilde{\varepsilon}. \tag{10}$$

In fact, from the above discussion case 2, we know that the inequality (10) always holds when $x^*(0) \in D_1$. Since $t_j - r_j$ is unbounded, we also can assume that it is increasing monotonically (we can realize this by choosing a subsequence) and $\lim_{j\to\infty} t_j - r_j = \infty$. So when $k > j$ and $0 \leq r \leq t_j - r_j$, we have

$$\min\{I_i^k(r_k + r)\} \leq \tilde{\varepsilon}, \quad i = T, V, B.$$

Now fix r and j and let $j \to \infty$. We have

$$\min\{I_i^*(r)\} = \lim_{k\to\infty} \min\{I_i^k(r_k + r)\} \leq \tilde{\varepsilon}, \quad i = T, V, B. \tag{11}$$

In fact, (11) holds for all $r \geq 0$ since $t_j - r_j$ is unbounded when j tends to infinity. This is contrary to (10). So D_2 is a uniform strong repeller for $\tilde{D}_1$. This finishes the proof.

Since $N_i \geq I_i, i = T, V, B$, then, from the strong uniform persistence of infection, we can get the strong uniform persistence of populations. Finally, we can obtain the strong uniform persistence of (1) relatively to all components.

Theorem 2 *When $R_0 > 1$, then there exists $\varepsilon > 0$ for system (1) such that, for any solutions $x(t)$ with initial values $N_T(0) > 0, N_V(0) > 0, N_B(0) > 0$ and $I_T(0) > 0$, $I_V(0) > 0$, or $I_B(0) > 0$, we have*

$$\lim_{t\to\infty} \inf \min\{I_T(t), N_T(t), I_V(t), N_V(t), I_B(t), N_B(t)\} > \varepsilon.$$

According to a general result from persistence theory, we can get the existence of the disease equilibrium for system (1).

Theorem 3 *When $R_0 > 1$, there exists at least one disease equilibrium of system (1).*

From the results above, we get that, when $R_0 > 1$, HIV will be spread in the MSM population so long as one infected individual is introduced in this population, regardless of whether he is an Only-Top, a Versatile or an Only-Bottom.

References

1. Thieme HR, **Persistence under relaxed point- dissipativity (with application to an endemic model)**. *SIAM J. Math. Anal.* 1993, **24**: 407–435.
